# Pellagra in Complex Clinical Settings: A Case Involving Bariatric Surgery, Whipple Procedure, and Alcohol Use Disorder

**DOI:** 10.7759/cureus.90935

**Published:** 2025-08-25

**Authors:** Kathryn C Lotharius, Gabrielle S Ferguson, Sebastian Camargo, George Luck, Parvathi Perumareddi

**Affiliations:** 1 College of Medicine, Florida Atlantic University Charles E. Schmidt College of Medicine, Boca Raton, USA; 2 Internal Medicine, Memorial Healthcare System, Pembroke Pines, USA; 3 Integrated Medical Science, Florida Atlantic University Charles E. Schmidt College of Medicine, Boca Raton, USA; 4 Family Medicine, Florida Atlantic University Charles E. Schmidt College of Medicine, Boca Raton, USA

**Keywords:** alcohol use disorder (aud), bariatric surgery complications, intestinal malabsorption, niacin deficiency, nutrition and dermatology, nutrition and metabolism, pellagra, whipple procedure complications

## Abstract

Pellagra is a disease of niacin (vitamin B3) deficiency and is classically characterized by the triad of diarrhea, dermatitis, and dementia, and has the potential to cause death. Pellagra can be caused by either insufficient dietary intake or dysfunctional utilization of niacin. Because niacin can be found in nearly every food group (meat, dairy, vegetables, grains, etc.), deficiency is rarely seen in resource-rich countries where food scarcity is less prevalent.

In this case, a 50-year-old woman presented to the emergency department with abdominal pain, watery diarrhea, altered mental status, altered taste, and a scaly rash on her upper extremities and perioral region. Her medical history was notable for alcohol use disorder, obesity previously treated with a gastric bypass, and chronic pancreatitis managed by a prior Whipple procedure. On examination, she was alert and oriented, with notable glossitis, cheilitis, excoriated rashes on the flexor surfaces of her hands, and 2+ pitting edema in both her upper and lower extremities. Laboratory findings were significant for megaloblastic anemia with a hemoglobin of 10.6 g/dL, hypoalbuminemia of 2.4 g/dL, transaminitis with alanine aminotransferase (ALT) of 60 units/L, and aspartate aminotransferase (AST) of 105 units/L, and deficiencies in niacin and vitamin D at less than 20 ng/mL and 12 ng/mL, respectively.

Given her clinical presentation, laboratory findings, and significant surgical and medical history, the patient was diagnosed with pellagra. Treatment was initiated with electrolyte and vitamin supplementation as well as furosemide to address the anasarca. The patient initially showed improvement with vitamin supplementation, and the edema in her upper extremities began to decrease. However, the patient developed acute changes in mental status, and a stroke alert was enacted. Although MRI and CT imaging revealed no acute intracranial abnormalities, elevated lactic acid levels (5.2 mmol/L) raised suspicion for seizure activity. She became encephalopathic and obtunded and was subsequently transferred to the ICU for further evaluation and management. Despite appropriate interventions, she continued to decompensate in the setting of hospital-acquired bacteremia and septic shock. She ultimately passed away one month later.

Although pellagra is a rare disease in the United States, it should be considered in patients presenting with the multi-system findings as seen in our patient. Bariatric and Whipple surgery patients are at particular risk for nutrient deficiencies, especially when compounded by alcohol use. It is crucial to educate patients who undergo bariatric or Whipple surgery on the importance of adhering to nutritional supplementation and limiting alcohol use, as these patients have the ideal conditions required for the development of pellagra. Niacin supplementation is the standard for treatment, and early intervention is key to preventing further complications and death.

## Introduction

Pellagra is a disease of niacin (vitamin B3) deficiency and is classically characterized by the triad of diarrhea, dermatitis, and dementia, and has the potential to cause death. Niacin is required for various steps of metabolism, including DNA repair and redox reactions, as it is needed to create nicotinamide adenine nucleotides (NAD) and nicotinamide adenine dinucleotide phosphate (NADP) [1]. Because niacin can be found in nearly every food group (meat, dairy, vegetables, grains, etc.), deficiency is rarely seen in resource-rich countries where food scarcity is less prevalent. Historically, pellagra reached epidemic proportions in the Southern United States in the early 1900s due to widespread poverty and diets made primarily of corn [2]. Currently, there are no national statistics readily available on the incidence and prevalence of pellagra, as the condition has become so rare and has only been documented in case reports rather than epidemiologic data sets [3]. Despite its rarity, pellagra can occur in the setting of alcohol use disorder, adverse medication (e.g., isoniazid) effects, genetic disease (e.g., Hartnup disease), or malabsorption [4]. In this case, we discuss a patient with a history of alcohol use disorder who had had extensive bariatric surgery and a Whipple procedure, who then subsequently developed pellagra.

## Case presentation

A 50-year-old woman with a past medical history of hypertension, alcohol use disorder, Bell’s palsy, obesity, recurrent pancreatitis, and previously treated syphilis presented to the emergency department (ED) with a five-day history of watery diarrhea. She reported experiencing generalized abdominal pain and occasional non-bloody diarrhea for the past four years. The patient also noted new symptoms, including paresthesias, episodes of forgetfulness, bilateral leg weakness, loss of balance, and altered taste/texture of her tongue over the past few weeks.

In the days leading up to her ED visit, she developed a scaly rash on her upper extremities. Upon presentation to the ED, she described diffuse, burning abdominal pain and reported having eight watery bowel movements per day. Review of systems was positive for weakness, nausea, and vomiting.

Her surgical history included a sleeve gastrectomy performed 19 years ago, which was later converted to a gastric bypass 16 years ago for obesity. She also underwent a Whipple procedure with Roux-en-Y gastrojejunostomy four years ago for chronic pancreatitis complicated by a pancreatic pseudocyst.

The patient was admitted to the hospital for further evaluation and management.

Labs revealed a low hemoglobin of 10.6 g/dL, an elevated mean corpuscular volume (MCV) of 99.7 fL, increased red cell distribution width (RDW) of 14.6 fL (Table 1), hypocalcemia of 7.7 mmol/L, hypoalbuminemia of 2.4 g/dL, hypoproteinemia of 5.7 g/dL, elevated alanine aminotransferase (ALT) of 60 units/L, elevated aspartate aminotransferase (AST) of 105 units/L, and hyperbilirubinemia of 1.4 mg/dL (Table 2). Her glucose level was elevated to 122 mg/dL in the non-fasting state, and a low anion gap was also noted of 3 mmol/L, suggesting alkalosis, and her alkaline phosphatase level was elevated to 162 units/L, likely attributed to her liver inflammation (Table 2).

**Table 1:** Complete Blood Count With Differential WBC, white blood cells; ANC, absolute neutrophil count; NRBC, nucleated red blood cells; MCV, mean corpuscular volume; MCH, mean corpuscular hemoglobin; MCHC, mean corpuscular hemoglobin concentration; MPV, mean platelet volume; Abs, antibodies.

Component	Value	Reference Range	Units
WBC	8.4	3.5-10.0	10^3^/μL
ANC (automated diff)	4.75	2.00-7.15	10^3^/μL
Auto NRBC%	0	≤0.0	
Red blood cell count	3.34 (Low)	4.00-5.50	Million/μL
Hemoglobin	10.6 (Low)	11.4-15.4	g/dL
Hematocrit	33.3	32.8-45.6	%
MCV	99.7 (High)	80.0-95.0	fL
MCH	31.7	26.0-34.0	pg
MCHC	31.8 (Low)	32.0-35.0	g/dL
Red cell distribution width	14.6 (High)	11.5-14.5	%
Platelets	353	150-450	10^3^/μL
MPV	11	9.4-12.4	fL
Neutrophils	56.5	42.5-73.2	%
Immature granulocytes %	0.4	0.0-0.6	%
Lymphocytes	37.6	18.2-47.4	%
Monocytes	4.9	4.3-11.0	%
Eosinophils	0.2	0.0-3.0	%
Neutrophil Abs	4.75	2.00-7.15	10^3^/μL
Lymphocyte Abs	3.16	1.16-3.18	10^3^/μL
Monocyte Abs	0.41	0.29-0.71	10^3^/μL
Eosinophil Abs	0.02 (Low)	0.03-0.27	10^3^/μL
Basophil Abs	0.03	0.01-0.05	10^3^/μL

**Table 2:** Comprehensive Metabolic Panel BUN, blood urea nitrogen; ALT, alanine aminotransferase; SGPT, serum glutamate pyruvate transaminase; AST, aspartate aminotransferase; SGOT, serum glutamic oxaloacetic transaminase; EGFR, estimated glomerular filtration rate.

Component	Value	Reference Range	Units
Glucose	122 (High)	74-99	mg/dL
BUN	12	7-17	mg/dL
Sodium	137	137-145	mmol/L
Potassium	3.6	3.5-5.1	mmol/L
Chloride	107	98-107	mmol/L
CO_2_	27	22-30	mmol/L
Anion gap	3 (Low)	5-15	mmol/L
Creatinine	0.66	0.51-0.95	mg/dL
Calcium	7.7 (Low)	8.4-10.2	mg/dL
Alkaline phosphatase	162 (High)	38-126	Units/L
Total protein	5.7 (Low)	6.3-8.2	g/dL
Albumin	2.4 (Low)	3.5-5.0	g/dL
ALT (SGPT)	60 (High)	≤35	Units/L
AST (SGOT)	105 (High)	15-46	Units/L
Bilirubin total	1.4 (High)	0.2-1.3	mg/dL
Race-neutral EGFR (CKD-EPI 2021)	107		mL/min/1.73 m^2^

Vitamin levels were significant for a low niacin level of less than 0.02 μg/mL, a low vitamin D level of less than 12 ng/mL, and an elevated methylmalonic acid level of 344 μmol/L. Of note, vitamin B12 levels and folate levels were within normal limits (Table 3). In addition, her stool culture was negative for bacteria, including *Clostridioides difficile*. Blood cultures revealed no growth at five days.

**Table 3:** Vitamins

Component	Value	Reference Range	Units
Riboflavin (Vitamin B2)	25.9	6.2-39.0	nmol/L
Niacin (Vitamin B3)	<0.02 (Low)	0.5-8.5	μg/mL
Folate (Vitamin B9)	18.2	2.76-20.00	ng/mL
Cobalamin (Vitamin B12)	740	239-931	pg/mL
Methylmalonic Acid	344 (High)	55-335	nmol/L
Homocysteine	5.22	3.70-13.90	μmol/L
Vitamin D 25 Hydroxy	<12 (Low)	30.0-100.0	ng/mL

**Figure 1:**
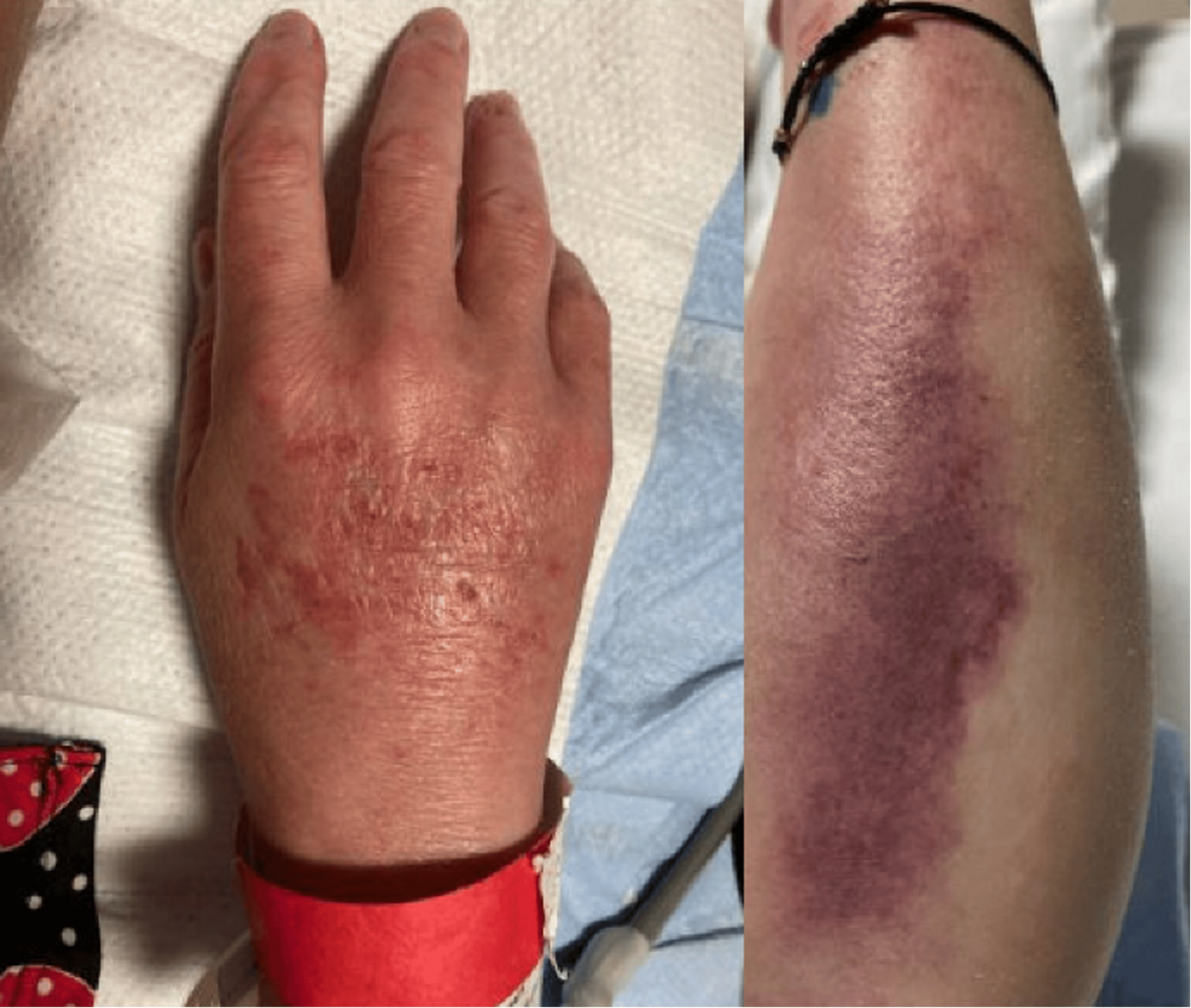
Photosensitive rash seen on the dorsum of hand and forearm

**Figure 2:**
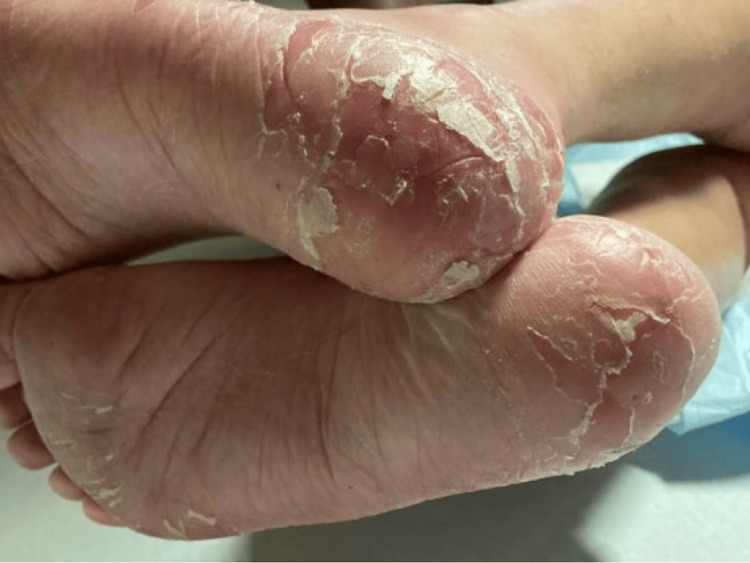
Desquamation seen on the heels

On physical examination, the patient was alert and oriented to person, place, and time. She was afebrile and hemodynamically stable with vital signs showing a blood pressure of 133/85 mmHg, heart rate of 89 bpm, respiratory rate of 18 bpm, and a temperature of 35.8°C. Notable findings included glossitis with a red, smooth tongue and cheilitis. Dermatologic examination revealed multiple excoriations in the flexor regions of both hands, as well as on the tips of her fingers. A photosensitive rash was observed on the dorsum of both hands and forearms (Figure 1), along with desquamation of the heels of both feet (Figure 2). There was also 2+ edema noted in both the upper and lower extremities. The patient's neurologic exam was within normal limits. 

The clinical presentation, laboratory findings, and significant surgical and medical history led to the diagnosis of pellagra. Treatment was initiated with electrolyte and vitamin supplementation, including niacin 500 mg PO daily, thiamine 100 mg PO daily, folic acid 1 mg PO daily, ferrous sulfate 325 mg PO daily, vitamin E 400 units daily, vitamin B6 25 mg IV daily, and vitamin D3 50,000 units weekly. Additionally, pancreatic enzyme replacement therapy (lipase, protease, and amylase) was administered twice daily. To address the anasarca, 40 mg of furosemide was given twice daily.

Clinical improvement was seen initially with vitamin supplementation, and the edema in her upper extremities began to decrease with furosemide. However, a stroke alert was called after the patient developed acute changes in mental status. Although MRI and CT imaging revealed no acute intracranial abnormalities, elevated lactic acid levels (5.2 mmol/L) raised suspicion for seizure activity. Other causes for her change in mental status were considered. At this stage, infection was deemed less likely due to her being afebrile with a normal white blood cell count. She became encephalopathic and obtunded and was subsequently transferred to the ICU for further evaluation and management. Despite urgent interventions, she continued to decompensate in the setting of hospital-acquired bacteremia and septic shock. She ultimately passed away one month later due to those complications.

## Discussion

The patient’s clinical picture most closely aligns with pellagra. The photosensitive rash on her hands and forearms and desquamation of her heels are characteristic skin findings. Her laboratory results proved her to be deficient in niacin, with a level of less than 0.02 μg/mL. One of the mechanisms by which this occurs is via decreased NAD+ production, a byproduct of niacin. Lower levels of NAD+ result in impaired tissue repair in the patient’s skin [5]. By a similar mechanism, her body’s inability to repair damaged intestinal mucosa due to niacin deficiency resulted in diarrhea. In the setting of alcohol use, accumulation of certain metabolites like 5-aminolevulinic acid (5-ALA) and kynurenic acid can contribute to the neuro-psychiatric features of pellagra [5]. Although our patient did not present with altered mental status on initial presentation, it cannot be ruled out that the seizure she had was related to pellagra. 

The history of extensive bariatric surgery, the Whipple procedure, and alcohol use disorder puts the patient at increased risk for the development of pellagra. In gastric bypass, meals bypass the duodenum, the primary location of niacin absorption (though some niacin is also absorbed in the jejunum) and enter directly into the jejunum from the stomach [6]. Although malabsorption is part of the mechanism by which patients lose weight via gastric bypass, it can also result in clinically significant nutrient deficiencies, including deficiencies in B vitamins and fat-soluble vitamins (vitamins A, D, E, and K). Additional factors include the patient’s history of Whipple procedure, in which the duodenum, head of the pancreas, gallbladder, and portion of the common bile duct are removed. This further contributes to malabsorption of nutrients via exocrine pancreatic insufficiency.

The role of chronic alcohol use in this patient’s disease progression is likely multifactorial. For instance, the presence of alcohol dehydrogenase (ADH) in the liver, the main enzyme responsible for alcohol metabolism, is decreased in patients with chronic hepatic dysfunction, which leads to increased blood alcohol levels and increased alcohol effects [7]. In the hepatic kynurenine pathway, tryptophan (a precursor of niacin) is degraded via the enzyme tryptophan 2,3-dioxygenase. Niacin is a byproduct of this pathway; however, alcohol inhibits this enzyme, thereby preventing niacin synthesis [8]. In addition, class I and class III ADH isozymes in the stomach contribute mildly to the first-pass metabolism of alcohol [9]. Further studies would need to be done to establish how the levels of gastric ADH are affected by the removal of portions of the stomach lining from gastric bypass. Chronic alcohol use also results in damage to the intestinal villi, which most likely contributed to further malabsorption in this patient [10]. The patient’s alcohol use disorder likely compounded the negative effects of her surgical procedures, which ultimately resulted in her diagnosis of pellagra.

## Conclusions

Although pellagra is a rare disease in developed countries, it is potentially fatal and should be considered in patients presenting with the multi-system findings seen in our patient. Patients who undergo bariatric and Whipple surgery are at particular risk for nutrient deficiencies, especially when compounded by alcohol use. It is crucial to educate patients who undergo bariatric or Whipple surgery on the importance of specific nutritional supplementation and limiting alcohol use, as these patients have the ideal conditions required for the development of pellagra. Niacin supplementation is the standard for treatment, and early intervention is key to preventing further complications and death.
